# The effect of HbA1C variability on the development and progression of diabetic nephropathy

**DOI:** 10.3389/fcdhc.2025.1718498

**Published:** 2025-12-01

**Authors:** Alper Coskun, Aslihan Calim, Emre Sedar Saygili, Tamer Sakaci, Feyza Yener Ozturk, Yener Koc, Fatih Borlu, Yuksel Altuntas, Taner Basturk

**Affiliations:** 1 Department of Internal Medicine, University of Health Sciences, Istanbul Sisli Hamidiye Etfal Training and Research Hospital, Istanbul, Türkiye; 2 Department of Endocrinology, University of Health Sciences, Istanbul Sisli Hamidiye Etfal Training and Research Hospital, Istanbul, Türkiye; 3 Department of Nephrology, University of Health Sciences, Istanbul Sisli Hamidiye Etfal Training and Research Hospital, Istanbul, Türkiye

**Keywords:** albuminuria, diabetic nephropathy, HbA1c, HbA1c variability, proteinuria

## Abstract

**Objective:**

Diabetes mellitus (DM) is a prevalent chronic disease that can lead to severe microvascular complications. Among these, diabetic nephropathy (DN) remains a leading cause of end-stage renal disease worldwide. Glycemic variability, reflecting fluctuations in blood glucose, has been suggested as a potential predictor of DM complications. This study aimed to investigate whether visit-to-visit HbA1c variability contributes to the development and progression of DN in patients with DM.

**Methods:**

In this retrospective cohort study, 228 patients were selected from 2,000 individuals diagnosed with DM between January 2007 and December 2017. A total of 80 patients without DN at baseline (ODN) and 148 patients with DN at baseline (WDN) were included in the study. HbA1c was measured 2–4 times per year over 3–5 years. Mean, standard deviation (SD), and coefficient of variation (CV) of HbA1c were calculated. Annual urea, creatinine, and albumin/protein levels were recorded. Logistic regression identified independent risk factors.

**Results:**

DN developed in 47 (58.8%) patients in the ODN group, whereas progression occurred in 44 (29.7%) patients in the WDN group. In the ODN group, higher HbA1c mean, SD, CV, hypertension, and albuminuria were significantly associated with DN onset (p<0.05). Logistic regression analysis confirmed HbA1c variability and hypertension as independent predictors. No significant association was found between HbA1c variability and DN progression.

**Conclusions:**

Variability in HbA1c is linked to the onset of DN but not its progression. These findings highlight the need for strategies targeting glycemic stability in DM management. Larger, multicenter prospective studies are warranted to confirm these results.

## Introduction

Diabetes mellitus (DM) is a long-term metabolic disorder that encompasses several subtypes and arises from a complex interaction of genetic predisposition and environmental influences. Among these, type 2 DM is the most prevalent, mainly due to impaired insulin sensitivity. The worldwide burden of DM continues to grow, driven largely by factors such as aging populations, rising obesity rates, sedentary lifestyles, and overall population expansion. Since the early 2000s, mortality attributable to DM has shown a steady upward trend, with approximately 1.6 million deaths directly linked to the disease in 2021 (1). The global prevalence was estimated at 10.5% in 2021 and is projected to increase to 12.2% by 2045 (2). The disease trajectory is frequently complicated by acute metabolic crises, including hypoglycemia and diabetic ketoacidosis, as well as chronic microvascular conditions such as retinopathy, diabetic nephropathy (DN), and neuropathy. Furthermore, macrovascular complications like coronary artery disease, stroke, and peripheral arterial disease play a major role in the elevated morbidity and mortality associated with DM.

DN, one of the major microvascular complications of DM, develops in approximately 20–40% of affected individuals (3) and was responsible for nearly 530,000 deaths worldwide in 2021 (1). Traditionally, DN is characterized by elevated urinary albumin excretion (UAE) often accompanied by hypertension, which progressively leads to a decline in glomerular filtration rate (GFR) and may ultimately progress to end-stage kidney disease (ESKD) (4). The main pathological alterations occur in the glomeruli, including thickening of the glomerular basement membrane, mesangial matrix expansion, glomerulosclerosis, and the formation of nodular (Kimmelstiel–Wilson) lesions, typically associated with hyaline deposition in arterioles (5, 6). Importantly, these histological features alone are not reliable predictors of prognosis. The clinical course of DN is generally divided into five stages: glomerular hyperfiltration with renal enlargement, a silent or latent period, early nephropathy marked by microalbuminuria, overt nephropathy characterized by albuminuria, and progression to ESKD. Both albuminuria and proteinuria remain well-recognized predictors of DN progression and ESKD risk, largely mediated by mechanisms such as upregulation of tubular chemokines, activation of the complement cascade, interstitial inflammation, and fibrotic remodeling (7). While albuminuria is an early marker of disease onset and progression, proteinuria continues to be considered a key indicator of severity and ongoing disease activity (8).

Hemoglobin A1c (HbA1c) is produced through the non-enzymatic glycation of the N-terminal valine of the hemoglobin beta chain (9), representing about 3–6% of the total hemoglobin content (10). Measurement of HbA1c is generally performed with either charge-based techniques, such as high-performance liquid chromatography (HPLC) and electrophoresis, or structure-specific methods, including affinity chromatography and immunoassays. Among these, HPLC is the most frequently applied (11). The coefficient of variation for HbA1c (HbA1c CV) in HPLC assays is typically maintained below 3.5% (12). HbA1c serves as a biomarker reflecting the average blood glucose level over a period of nearly three months, with roughly half of its value influenced by the preceding month and the remainder by the prior 2–4 months (13). Results from the Diabetes Control and Complications Trial (DCCT) indicated that each 1% increase in HbA1c corresponded to an approximate 35 mg/dL rise in mean plasma glucose. Moreover, the DCCT showed that lowering HbA1c by 1% in patients with type 1 DM significantly reduced the risk of retinopathy by 35%, DN by 24–44%, and neuropathy by 30% (14). Consistently, findings from the United Kingdom Prospective Diabetes Study (UKPDS) demonstrated that in individuals with type 2 DM, a 1% reduction in HbA1c was associated with a 25% decrease in DM-related mortality, a 7% decline in all-cause mortality, an 18% reduction in myocardial infarction, and a 35% decrease in microvascular complications (15).

DCCT first introduced the concept of glycemic variability, describing it as the standard deviation of mean glucose values assessed every three months (16). Glycemic variability includes both short-term fluctuations in blood glucose that occur within or between days, and long-term changes in HbA1c measured over weeks to months (17). While HbA1c provides an estimate of overall glycemic control, it does not reflect daily glucose swings or hypoglycemic episodes. Evidence suggests that abrupt alterations in glucose levels can trigger oxidative stress and promote microvascular injury independently of sustained hyperglycemia. Elevated glycemic variability has also been linked with an increased likelihood of hypoglycemia. Moreover, both hyperglycemic excursions and hypoglycemic events may activate inflammatory, oxidative, and prothrombotic pathways, thereby contributing to the development and progression of DM-related complications.

Findings from the DCCT showed that, despite having comparable mean HbA1c values, individuals managed with conventional treatment exhibited higher rates of retinopathy than those receiving intensive therapy (18). This observation highlights that factors other than average HbA1c contribute to the risk of complications. Later investigations in both type 1 and type 2 DM further demonstrated that fluctuations in HbA1c are independently associated with the occurrence of microvascular complications, regardless of mean HbA1c levels (19–22). Consequently, glycemic variability has emerged as an additional dimension of glycemic control and may serve as a more informative predictor of DM complications than mean HbA1c alone. Building on this rationale, the present study was designed to investigate the effect of HbA1c variability on the onset and progression of DN.

## Materials and methods

### Study design and population

This retrospective analysis included medical records of 2,000 individuals who visited the Internal Medicine, Nephrology, or Endocrinology outpatient clinics at the University of Health Sciences, Istanbul Şişli Hamidiye Etfal Training and Research Hospital, between January 1, 2007, and December 30, 2017. Eligibility required a diagnosis of DM based on the American Diabetes Association (ADA) criteria and the availability of sufficient clinical and laboratory information. After applying the inclusion and exclusion criteria, 228 patients with type 2 DM were ultimately included in the study.

The inclusion criteria were as follows: confirmed diagnosis of type 2 DM, follow-up visits every 3–6 months for at least three years, availability of serial measurements of HbA1c, LDL cholesterol, serum urea and creatinine, as well as spot or 24-hour urine albumin/protein levels, and age ≥18 years. Exclusion criteria consisted of type 1 DM, age <18 years, pregnancy, advanced chronic liver disease, estimated glomerular filtration rate (eGFR) <30 mL/min/1.73 m² (calculated using the Modification of Diet in Renal Disease [MDRD] equation), history of solid or hematologic malignancy, active or past glomerulonephritis, hemoglobinopathies or other hemolytic anemias, prior kidney transplantation, renal replacement therapy, or immunosuppressive treatment. The diagnosis of DN was based on clinical and laboratory findings, as data regarding renal biopsy were not available in this retrospective study.

At study entry, patients were divided into two cohorts based on the presence or absence of DN: 80 patients without DN at baseline (ODN) and 148 patients with DN at baseline (WDN).

### Data collection

Baseline demographic and clinical features, comorbidities, and treatment regimens—particularly the use of antihypertensive medications—were documented. Laboratory data, including serum urea, creatinine, and urinary albumin/protein levels (from spot or 24-hour urine collections), were obtained annually over a follow-up period of 3 to 5 years. HbA1c values, measured between two and four times per year, were retrieved from the hospital’s electronic medical records.

eGFR was determined using the MDRD equation: eGFR= 186 x serum creatinine - 1.154 x age - 0.203 x [0.742 if female] x [1.21 if black]. For each participant, mean HbA1c (HbA1c Mean), standard deviation (HbA1c SD), and HbA1c CV were calculated based on HbA1c values measured at intervals of at least three months. HbA1c CV was derived using the formula: HbA1c SD/HbA1c Mean.

### Laboratory methods

Venous blood samples were collected after at least 12 hours of overnight fasting. Serum urea and creatinine concentrations were determined using a Cobas E analyzer. Urinary albumin and protein levels, obtained either from spot urine samples (microalbumin/creatinine ratio) or 24-hour urine collections, were analyzed with a Siemens Nephelometer BN ProSpec system. HbA1c levels were measured by HPLC on a Tosoh G-8 analyzer, with an intra-assay CV maintained below 3.5%.

### Definitions

The development of DN was defined by the presence of UAE ≥30 mg/day, urinary protein excretion ≥150 mg/day, and/or an eGFR below 90 mL/min/1.73 m². Progression of DN was determined as either an advancement in chronic kidney disease (CKD) stage or a reduction of ≥25% from baseline eGFR. CKD stages were classified according to the Kidney Disease: Improving Global Outcomes (KDIGO) 2002 criteria (23). At study entry, among the 148 patients with DN, 45 were identified as stage 1 CKD, 53 as stage 2 CKD, and 50 as stage 3 CKD.

During the retrospective assessment of DN development and progression from hospital electronic records, patients with infection, sepsis, recent (within one month) surgical procedures, or fever related to acute or chronic conditions were identified, and any albuminuria, proteinuria, or eGFR decline associated with these situations were excluded from the analysis.

### Statistical analysis

All statistical analyses were carried out using SPSS software, version 15.0 for Windows. Continuous variables were summarized as mean, median, standard deviation, minimum, and maximum values. For comparisons between two independent groups, the Student’s t-test was applied when variables followed a normal distribution, whereas the Mann–Whitney U test was used for non-normally distributed data. Categorical variables were analyzed with the Chi-square test. To determine independent predictors, logistic regression analysis was employed. A p-value of <0.05 was considered indicative of statistical significance.

### Ethics statement

This study received approval from the Clinical Research Ethics Committee of the General Secretariat of the Istanbul Beyoğlu Public Hospitals Association, Şişli Hamidiye Etfal Training and Research Hospital (Approval No: 1433; date: March 7, 2017).

## Results

In the ODN group, the mean age was 57.6 ± 8.4 years, and 51 participants (63.8%) were female. The mean follow-up duration for the ODN group was 3.9 ± 0.8 years. In the WDN group, the mean age was 62.5 ± 7.5 years, and 91 patients (61.5%) were female. The mean follow-up duration for the WDN group was 4.0 ± 0.8 years. The demographic, clinical, and laboratory characteristics of both groups are summarized in **Table 1**.

**Table 1:** The general characteristics of patients.

Characteristics		ODN (n=80)	WDN (n=148)
Age		57.6 ± 8.4 (34-73)	62.5 ± 7.5 (45-80)
Sex	Male	29 (36.3)	57 (38.5)
	Female	51 (63.8)	91 (61.5)
Smoking (package/year)		13.2 ± 26.2 (0-140)	8.5 ± 20.3 (0-120)
BMI (kilograms/square meter)		30.4 ± 4.7 (22.4-48)	32.2 ± 7.5 (19.9-75)
Duration of DM (years)		13.4 ± 7.6 (3-47)	16.8 ± 7.4 (4-43)
Hypertension		53 (66.3)	129 (87.2)
Hyperlipidemia		55 (68.8)	90 (60.8)
IHD		13 (16.3)	39 (26.4)
PAD		1 (1.3)	5 (3.4)
CVA		0 (0.0)	5 (3.4)
ACE-I/ARB		49 (61.3)	115 (77.7)
Urea (milligrams/deciliter)		28.7 ± 7.6 (15-49)	38.2 ± 14.5 (14-87)
Creatinine (milligrams/deciliter)		0.68 ± 0.12 (0.5-0.91)	0.99 ± 0.33 (0.4-2.17)
Proteinuria (milligrams/day)		91.8 ± 28.7 (40.9-148.1)	531.2 ± 988.0 (6.5-5822)
Albuminuria (milligrams/day)		11.1 ± 7.1 (2.4-29.3)	283.1 ± 731.3 (4.1-4907.4)
eGFR (milliliters/minute/1.73 square meter)		109.3 ± 15.0 (90-171)	78.1 ± 29.2 (30-184)
Number of HbA1C Measurements		7.2 ± 2.4 (4-15)	8.3 ± 2.6 (4-15)
HbA1C Mean		9.12 ± 2.05 (5.56-15.6)	9.06 ± 1.77 (5.80-14.29)
HbA1C SD		1.21 ± 0.91 (0.1-4.3)	1.10 ± 0.67 (0.19-4.33)
HbA1C CV		0.13 ± 0.09 (0.01-0.48)	0.12 ± 0.07 (0.03-0.41)

ACE-I, angiotensin-converting enzyme inhibitors; ARB, angiotensin-II receptor blockers; BMI, body mass index; DN, diabetic nephropathy; CV, coefficient of variation; CVA, cerebrovascular accident; DM, diabetes mellitus; eGFR, estimated glomerular filtration rate; IHD, ischemic heart disease; ODN, patients without DN at baseline; PAD, peripheral artery disease; SD, standard deviation; WDN, patients with DN at baseline.

Continuous variables are presented as mean ± SD (minimum-maximum) and categorical variables as number and percentages.

During the follow-up period, DN developed in 47 patients (58.8%) from the ODN group. The majority of cases emerged in the second (n=25, 53.2%) and third (n=20, 42.6%) years, while only two patients (4.3%) were diagnosed in the fourth year. Of these, 33 patients (41.3%) presented with DN characterized by albuminuria and/or proteinuria, whereas 14 patients (17.5%) exhibited DN defined by a reduction in eGFR without concurrent albuminuria or proteinuria. In this latter subgroup, baseline eGFR averaged 101.3 ± 10.2 mL/min, declining to 84.7 ± 4.5 mL/min at the time of DN diagnosis. In the WDN group, baseline kidney function showed that 45 patients (30.4%) had Stage 1 CKD, 53 (35.8%) had Stage 2, and 50 (33.8%) had Stage 3 disease. During follow-up, 44 patients (29.7%) experienced DN progression, occurring most frequently in the second (n=17, 38.6%) and third (n=14, 31.8%) years, followed by the fourth (n=10, 22.7%) and fifth (n=3, 6.8%) years. At baseline, albuminuria and/or proteinuria was observed in 98 patients (66.2%), while 27 patients (18.2%) remained free of these findings throughout follow-up. The mean number of HbA1c assessments was 7.2 ± 2.4 in the ODN group compared with 8.3 ± 2.6 in the WDN group.

In the ODN group, patients who developed DN during the follow-up period exhibited higher rates of hypertension (p=0.001), elevated albuminuria levels (p=0.022), and significantly greater HbA1c SD (p=0.019), HbA1c Mean (p=0.038), and HbA1c CV (p=0.034) values compared with those who did not develop DN. These findings are summarized in **Table 2**.

**Table 2:** Follow-up data of ODN group.

ODN (N = 80)			
Characteristics	Development of DN During Follow-up		
	No (n=33)	Yes (n=47)	p
Age	55.8 ± 9.4	58.9 ± 7.5	0.118
Sex			0.056
Female	17 (51.5%)	34 (72.3%)	
Male	16 (48.5%)	13 (27.7%)	
Smoking Status	11 (44.0%)	12 (36.4%)	0.556
Smoking (pack years)	8.5 ± 13.1	16.7 ± 32.7	0.950
BMI (kilograms/square meter)	29.6 ± 3.3	30.9 ± 5.6	0.276
Duration of DM (years)	13.1 ± 8.5	13.5 ± 7.0	0.553
Hypertension	**15 (45.5%)**	**38 (80.9%)**	**0.001**
Hyperlipidemia	21 (63.6%)	34 (72.3%)	0.408
IHD	4 (12.1%)	9 (19.1%)	0.402
PAD	0 (0.0%)	1 (2.1%)	1.000
ACE-I/ARB	17 (51.5%)	32 (68.1%)	0.134
Urea (milligrams/deciliter)	26.9 ± 6.3	30.0 ± 8.1	0.117
Creatinine (milligrams/deciliter)	0.71 ± 0.11	0.67 ± 0.12	0.118
Proteinuria (milligrams/day)	84.0 ± 26.7	96.0 ± 29.5	0.268
Albuminuria (milligrams/day)	**9.4 ± 7.0**	**12.3 ± 7.1**	**0.022**
eGFR (milliliters/minute/1.73 square meter)	109.7 ± 16.2	109.0 ± 14.2	0.844
HbA1c SD	**0.92 ± 0.66**	**1.41 ± 1.02**	**0.019**
HbA1c SD IQR			0.153
<0.5925	13 (39.4%)	11 (23.4%)	
0.5925-0.9660	7 (21.2%)	6 (12.8%)	
0.9660-1.4943	7 (21.2%)	12 (25.5%)	
>1.4943	6 (18.2%)	18 (38.3%)	
HbA1c Mean	**8.56 ± 2.05**	**9.52 ± 1.98**	**0.038**
HbA1c Mean IQR			0.053
<7.6355	13 (39.4%)	8 (17.0%)	
7.6355-8.9085	10 (30.3%)	11 (23.4%)	
8.9085-10.3123	5 (15.2%)	12 (25.5%)	
>10.3123	5 (15.2%)	16 (34.0%)	
HbA1c CV	**0.10 ± 0.06**	**0.15 ± 0.10**	**0.034**
HbA1c CV IQR			0.239
<0.0713	11 (33.3%)	10 (21.3%)	
0.0713-0.1080	10 (30.3%)	9 (19.1%)	
0.1080-0.1510	6 (18.2%)	13 (27.7%)	
>0.1510	6 (18.2%)	15 (31.9%)	

ACE-I, angiotensin converting enzyme inhibitor; ARB, angiotensin II receptor blocker; BMI, body mass index; CV, coefficient of variation; DM, diabetes mellitus; DN, diabetic nephropathy; eGFR, estimated glomerular filtration rate; IHD, ischemic heart disease; IQR, interquartile range; ODN, patients without DN at baseline; PAD, peripheral artery disease; SD, standard deviation.

Continuous variables are presented as mean ± SD and categorical variables as number and percentages.

Values in bold indicate statistical significance (p < 0.05).

In the WDN group, patients who experienced DN progression had significantly higher frequencies of male sex (p=0.009), smoking status (p=0.012), cigarette pack-years (p=0.010), proteinuria (p=0.001), and albuminuria (p=0.006) compared with those without progression. These results are summarized in **Table 3**.

**Table 3:** Follow-up data of WDN group.

WDN (N = 148)			
Characteristics	Progression of DN During Follow-up		
	No (n=104)	Yes (n=44)	p
Age	62.6 ± 7.9	62.1 ± 6.2	0.819
Sex			**0.009**
Female	**71 (68.3%)**	**20 (45.5%)**	
Male	**33 (31.7%)**	**24 (54.5%)**	
Smoking Status	**10 (16.9%)**	**10 (43.5%)**	**0.012**
Smoking (pack years)	**6.0 ± 19.8**	**14.9 ± 20.5**	**0.010**
BMI (kilograms/square meter)	31.4 ± 6.2	34.1 ± 9.7	0.127
Duration of DM (years)	17.0 ± 7.6	16.3 ± 7.1	0.663
Hypertension	90 (86.5%)	39 (88.6%)	0.727
Hyperlipidemia	63 (60.6%)	27 (61.4%)	0.929
IHD	27 (26.0%)	12 (27.3%)	0.869
PAD	4 (3.8%)	1 (2.3%)	1.000
CVA	3 (2.9%)	2 (4.5%)	0.634
ACE-I/ARB	84 (80.8%)	31 (70.5%)	0.168
Urea (milligrams/deciliter)	37.7 ± 14.3	39.2 ± 15.0	0.680
Creatinine (milligrams/deciliter)	0.98 ± 0.32	1.01 ± 0.37	0.988
Proteinuria (milligrams/day)	**288.7 ± 319.8**	**1115.3 ± 1637.2**	**0.001**
Albuminuria (milligrams/day)	**156.2 ± 322.4**	**659.2 ± 1,291.2**	**0.006**
eGFR (milliliters/minute/1.73 square meter)	77.6 ± 30.4	79.2 ± 26.2	0.762
HbA1c SD	1.04 ± 0.60	1.25 ± 0.80	0.162
HbA1c SD IQR			0.399
<0.5925	27 (26.0%)	6 (13.6%)	
0.5925-0.9660	30 (28.8%)	14 (31.8%)	
0.9660-1.4943	26 (25.0%)	12 (27.3%)	
>1.4943	21 (20.2%)	12 (27.3%)	
HbA1c Mean	8.88 ± 1.80	9.50 ± 1.64	0.051
HbA1c Mean IQR			0.186
<7.6355	29 (27.9%)	7 (15.9%)	
7.6355-8.9085	27 (26.0%)	9 (20.5%)	
8.9085-10.3123	27 (26.0%)	13 (29.5%)	
>10.3123	21 (20.2%)	15 (34.1%)	
HbA1c CV	0.12 ± 0.06	0.13 ± 0.08	0.340
HbA1c CV IQR			0.919
<0.0713	27 (26.0%)	10 (22.7%)	
0.0713-0.1080	27 (26.0%)	11 (25.0%)	
0.1080-0.1510	27 (26.0%)	11 (25.0%)	
>0.1510	23 (22.1%)	12 (27.3%)	

ACE-I, angiotensin converting enzyme inhibitor; ARB, angiotensin II receptor blocker; BMI, body mass index; CV, coefficient of variation; CVA, cerebrovascular accident; DM, diabetes mellitus; DN, diabetic nephropathy; eGFR, estimated glomerular filtration rate; IHD, ischemic heart disease; IQR, interquartile range; PAD, peripheral artery disease; SD, standard deviation; WDN, patients with DN at baseline.

Continuous variables are presented as mean ± SD and categorical variables as number and percentages.

Values in bold indicate statistical significance (p < 0.05).

Univariate logistic regression analysis revealed that in the ODN group, hypertension (p=0.001), HbA1c SD (p=0.025), HbA1c Mean (p=0.043), and HbA1c CV (p=0.046) were significant predictors of DN development. In the WDN group, male sex (p=0.010), smoking status (p=0.015), proteinuria (p=0.017), albuminuria (p=0.029), and HbA1c Mean IQR>10.31 (p=0.045) emerged as significant predictors of DN progression. The results of these analyses are presented in **Table 4**.

**Table 4:** Univariate logistic regression analysis of risk factors for the development and progression of DN.

Factors	ODN group (n = 80)				WDN group (n = 148)			
	OR	95% CI		p	OR	95% CI		p
		Min	Max			Min	Max	
Age	1.048	0.991	1.107	0.100	0.990	0.944	1.038	0.667
Sex	2.462	0.966	6.271	0.059	**0.387**	**0.188**	**0.798**	**0.010**
Smoking Status	0.727	0.252	2.102	0.557	**3.769**	**1.295**	**10.975**	**0.015**
Smoking (pack year)	1.015	0.989	1.041	0.262	1.020	0.996	1.044	0.100
BMI	1.066	0.948	1.200	0.287	1.048	0.993	1.106	0.087
Duration of DM (years)	1.008	0.950	1.069	0.798	0.985	0.936	1.037	0.576
Hypertension	**5.067**	**1.866**	**13.755**	**0.001**	1.213	0.409	3.602	0.728
Hyperlipidemia	1.495	0.575	3.883	0.409	1.034	0.502	2.130	0.929
IHD	1.717	0.481	6.133	0.405	1.069	0.483	2.369	0.869
PAD	1.000				0.581	0.063	5.355	0.632
CVA					1.603	0.258	9.944	0.612
ACE-I/ARB	2.008	0.802	5.027	0.137	0.568	0.252	1.277	0.171
Urea	1.060	0.990	1.134	0.094	1.007	0.982	1.032	0.594
Creatinine	0.058	0.001	2.819	0.151	1.313	0.462	3.737	0.609
Proteinuria	1.016	0.988	1.045	0.262	**1.002**	**1.000**	**1.003**	**0.017**
Albuminuria	1.067	0.990	1.149	0.089	**1.001**	**1.000**	**1.002**	**0.029**
eGFR	0.997	0.968	1.027	0.842	1.002	0.990	1.014	0.761
HbA1c SD	**2.059**	**1.096**	**3.869**	**0.025**	1.573	0.940	2.631	0.085
HbA1c SD IQR (Ref: <0.5925)				0.166				0.414
0.5925-0.9660	1.013	0.262	3.924	0.985	2.100	0.707	6.237	0.182
0.9660-1.4943	2.026	0.592	6.933	0.261	2.077	0.679	6.354	0.200
>1.4943	**3.545**	**1.042**	**12.058**	**0.043**	2.571	0.827	7.991	0.103
HbA1c Mean	**1.287**	**1.008**	**1.644**	**0.043**	1.219	0.997	1.490	0.054
HbA1c Mean IQR (Ref: <7.6355)				0.064				0.197
7.6355-8.9085	1.788	0.523	6.106	0.354	1.381	0.451	4.225	0.572
8.9085-10.3123	3.900	0.996	15.276	0.051	1.995	0.693	5.745	0.201
>10.3123	**5.200**	**1.367**	**19.774**	**0.016**	**2.959**	**1.027**	**8.528**	**0.045**
HbA1c CV	**1.067**	**1.001**	**1.138**	**0.046**	1.035	0.984	1.089	0.183
HbA1c CV IQR (Ref: <0.0713)				0.248				0.919
0.0713-0.1080	0.990	0.286	3.430	0.987	1.100	0.401	3.017	0.853
0.1080-0.1510	2.383	0.655	8.675	0.188	1.229	0.448	3.264	0.719
>0.1510	2.750	0.767	9.857	0.120	1.409	0.515	3.855	0.505

ACE-I, angiotensin converting enzyme inhibitor; ARB, angiotensin II receptor blocker; BMI, body mass index; CI, confidence interval; CV, coefficient of variation; CVA, cerebrovascular accident; DM, diabetes mellitus; DN, diabetic nephropathy; eGFR, estimated glomerular filtration rate; IHD, ischemic heart disease; IQR, interquartile range; ODN, patients without DN at baseline; OR, odds ratio; PAD, peripheral artery disease; SD, standard deviation; WDN, patients with DN at baseline.

Values in bold indicate statistical significance (p < 0.05).

A predictive model incorporating these potential risk factors was further tested with multivariate logistic regression. This analysis identified HbA1c SD as an independent risk factor for DN development in the ODN group (p=0.025, Forward method). The results of the multivariate logistic regression are presented in **Supplementary Table S1**.

## Discussion

In this study assessing the impact of HbA1c variability on DN, we found that increased HbA1c variability was associated with DN development in patients without DN at baseline (ODN group). Conversely, in patients who already had DN at baseline (WDN group), HbA1c variability did not significantly affect DN progression.

HbA1c variability is increasingly acknowledged as a robust predictor of DM complications, offering insight into long-term glycemic control beyond average HbA1c levels. Gorst et al. reported that higher HbA1c variability is associated with microvascular and macrovascular complications, as well as increased mortality, independent of HbA1c Mean (17). Similarly, the Renal Insufficiency And Cardiovascular Events (RIACE) study demonstrated that HbA1c variability affected albuminuria and CKD phenotypes regardless of HbA1c Mean (24). Other studies have consistently identified HbA1c variability as an independent risk factor for DN onset and progression (25–27), including in type 1 DM (28, 29). Our findings align with these results, confirming that HbA1c Mean, HbA1c SD, and HbA1c CV are significant predictors of DN development in ODN group.

In a cohort of type 2 DM patients, Rodríguez-Segade et al. found DN incidence to be 18.3%, with HbA1c variability as an independent risk factor (30). The higher rate in our study (58.8%) may stem from differing DN definitions, as Rodríguez-Segade et al. used only 24-hour UAE. Low et al. showed that 75% of normoalbuminuric patients who developed DN had albuminuria without eGFR decline, while 25% had isolated eGFR reduction (31). Similarly, in our cohort, 17.5% developed DN characterized by eGFR decline without albuminuria or proteinuria. These findings underscore the importance of assessing both serum creatinine/eGFR and albumin-to-creatinine ratio (ACR) in DN monitoring, as recommended by the ADA 2018 guidelines (32).

Interestingly, contrary to some previous studies, HbA1c Mean, HbA1c SD, and HbA1c CV were not predictive of DN progression in WDN group in our study. This may be explained by differences in endpoint definitions-while Low et al. defined progression as eGFR <60 mL/min (31), we defined it as advancement to a higher CKD stage. Such methodological variations likely contributed to the observed discrepancies.

Studies conducted in China and Taiwan have highlighted the importance of postprandial glucose variability in addition to HbA1c variability. Both measures were strongly associated with DN and other microvascular complications (33, 34). *Post hoc* analyses revealed that combined variability in HbA1c and postprandial glucose increased the risk of microvascular complications, cardiovascular events, stroke, and all-cause mortality (35). Dorajoo et al. demonstrated that combining HbA1c Mean, baseline ACR, HbA1c CV, and hypertension enhanced the prediction of new-onset albuminuria in normoalbuminuric type 2 DM patients (36). Similarly, Ceriello et al. reported that, in addition to HbA1c variability, fluctuations in systolic and diastolic blood pressure, lipid levels, and uric acid contributed to DN risk. Specifically, higher HDL cholesterol variability combined with elevated HbA1c variability was most strongly associated with microalbuminuria, while variability in uric acid and diastolic blood pressure correlated most with eGFR decline (37). These findings suggest that assessing HbA1c variability alongside other clinical parameters, especially postprandial glucose fluctuations, can improve DN risk evaluation.

Further evidence from a cohort of 855 intensively treated type 2 DM patients in China indicated that HbA1c variability was linked to major microvascular events. Notably, patients with higher HbA1c variability experienced more frequent DN development and progression, even among those with good glycemic control (HbA1c ≤ 7.0%) (38). This supports the notion that glycemic fluctuations and hypoglycemic episodes may drive DN onset and progression despite overall adequate glucose management.

HbA1c levels are influenced not only by plasma glucose concentrations but also by various hematologic and metabolic conditions that alter erythrocyte turnover. In hemolytic anemia, accelerated red cell destruction can result in falsely low HbA1c values, whereas conditions such as uremia or iron deficiency anemia may lead to falsely elevated values due to reduced erythrocyte turnover (39, 40). In patients with CKD, carbamylated hemoglobin may also contribute to spuriously high HbA1c results, while shortened erythrocyte lifespan, the use of erythropoiesis-stimulating agents, or blood transfusions can lead to underestimation of HbA1c (41). Furthermore, elevated fetal hemoglobin levels in hemoglobinopathies such as thalassemia major or intermedia may cause falsely low HbA1c measurements (42). In our study, patients with hemoglobinopathies (e.g., thalassemia major, thalassemia minor) and those with active or previous hemolytic anemia were excluded. However, data regarding the presence or absence of iron, vitamin B12, and folic acid deficiency anemia, as well as anemia of chronic disease, were not available. This limitation should be taken into consideration when interpreting our findings, as these factors may have influenced HbA1c levels independently of glycemic status.

Sex and smoking status also appear to influence DN progression. Male sex has been identified as an independent risk factor for eGFR decline (43) and faster progression to dialysis (44). In line with these reports, our study found male sex to be a significant predictor of DN progression in the WDN group. Similarly, smoking has been associated with increased DN risk: a meta-analysis demonstrated higher DN incidence among current smokers compared to never-smokers (45), and both smoking duration and cumulative pack-years have been shown to predict DN progression independently (46). Consistent with these findings, smoking and pack-years were significant risk factors in our cohort.

Given the accumulating evidence linking HbA1c variability with microvascular and macrovascular complications, interventions to reduce glycemic fluctuations have gained attention. In a study of approximately 20,000 patients with type 2 DM, sodium-glucose cotransporter 2 (SGLT2) inhibitors were more effective than dipeptidyl peptidase-4 (DPP-4) inhibitors in reducing HbA1c variability and were associated with fewer major cardiovascular and renal events (47). Another study reported that SGLT2 inhibitors improved both diabetic neuropathy and DN outcomes (48). These observations underscore the potential clinical value of targeting glycemic variability in addition to reducing mean HbA1c levels.

This study has several limitations. First, it was a single-center, retrospective study, which may limit generalizability. Second, according to the 2012 KDIGO guidelines, CKD is defined as eGFR < 60 mL/min and/or ACR > 30 mg/g persisting for at least three months (49). Because we could not confirm persistence of these findings, DN development or progression may have been overestimated. Third, eGFR was calculated using the MDRD equation, which can underestimate renal function in patients with normal or near-normal eGFR (50–52). Nonetheless, MDRD has shown comparable or superior accuracy to Chronic Kidney Disease Epidemiology Collaboration (CKD-EPI) in patients with DM and eGFR <60 mL/min (53). Data on renal biopsy were unavailable, preventing exclusion of alternative diagnoses such as FSGS. Finally, the absence of data on postprandial glucose variability—a known independent risk factor for DN progression— represents another limitation of this retrospective study.

## Conclusion

Our findings indicate that HbA1c CV is a significant predictor of albuminuria and/or eGFR decline in ODN group. Alongside established risk factors such as HbA1c Mean, UAE, and hypertension, HbA1c variability emerges as an important contributor to DN development. Monitoring HbA1c variability may therefore provide valuable insights for optimizing glycemic targets in type 2 DM, identifying patients at elevated risk for DN earlier, and guiding timely preventive interventions.

## Data Availability

The raw data supporting the conclusions of this article will be made available by the authors, without undue reservation.
